# The Importance of Protein Phosphorylation for Signaling and Metabolism in Response to Diel Light Cycling and Nutrient Availability in a Marine Diatom

**DOI:** 10.3390/biology9070155

**Published:** 2020-07-06

**Authors:** Maxine H. Tan, Sarah R. Smith, Kim K. Hixson, Justin Tan, James K. McCarthy, Adam B. Kustka, Andrew E. Allen

**Affiliations:** 1 Scripps Institution of Oceanography, Integrative Oceanography Division, University of California, San Diego, La Jolla, CA 92037, USA; mht005@ucsd.edu; 2 Microbial and Environmental Genomics, J. Craig Venter Institute, La Jolla, CA 92037, USA; Sarah.Smith@jcvi.org (S.R.S.); jmccarth@jcvi.org (J.K.M.); 3 Environmental Molecular Sciences Laboratory, Pacific Northwest National Laboratory, Richland, WA 99354, USA; kim.hixson@pnnl.gov; 4 Department of Bioengineering, University of California, San Diego, La Jolla, CA 92037, USA; tjustin@ucsd.edu; 5 Department of Earth and Environmental Sciences, Rutgers University, Newark, NJ 07102, USA; kustka@newark.rutgers.edu

**Keywords:** phosphoproteome, nitrogen stress, iron, diel, diatom, signaling

## Abstract

Diatoms are major contributors to global primary production and their populations in the modern oceans are affected by availability of iron, nitrogen, phosphate, silica, and other trace metals, vitamins, and infochemicals. However, little is known about the role of phosphorylation in diatoms and its role in regulation and signaling. We report a total of 2759 phosphorylation sites on 1502 proteins detected in *Phaeodactylum tricornutum*. Conditionally phosphorylated peptides were detected at low iron (*n* = 108), during the diel cycle (*n* = 149), and due to nitrogen availability (*n* = 137). Through a multi-omic comparison of transcript, protein, phosphorylation, and protein homology, we identify numerous proteins and key cellular processes that are likely under control of phospho-regulation. We show that phosphorylation regulates: (1) carbon retrenchment and reallocation during growth under low iron, (2) carbon flux towards lipid biosynthesis after the lights turn on, (3) coordination of transcription and translation over the diel cycle and (4) in response to nitrogen depletion. We also uncover phosphorylation sites for proteins that play major roles in diatom Fe sensing and utilization, including flavodoxin and phytotransferrin (ISIP2A), as well as identify phospho-regulated stress proteins and kinases. These findings provide much needed insight into the roles of protein phosphorylation in diel cycling and nutrient sensing in diatoms.

## 1. Introduction

Widely distributed across the world’s aquatic environments, diatoms are important unicellular eukaryotic phytoplankton that are responsible for approximately 20% of annual global primary production and play a significant role in biogeochemical cycles [1]. The availability of nutrients such as iron (Fe), nitrogen (N), and phosphorus, is essential for the rapid rise and fall of diatom populations [2,3]. Diatom population dynamics, affected by the periodic supply of dissolved Fe and N, have been studied in high-nutrient, low chlorophyll (HNLC) regions [4,5,6], along upwelling coasts [7,8], and in polar regions [9].

In response to low Fe conditions, diatoms have been noted to resort to retrenchment and reallocation of N and carbon (C), drastically decreasing NO_3_ and increasing NH_4_ assimilation pathways while also increasing Fe scavenging proteins and reducing Fe-containing proteins [10,11,12,13]. Physiological characteristics of these cells include low chlorophyll content, low photosynthetic efficiency, and decreased growth rate. Prolonged exposure to low Fe causes cells to rearrange their bioenergetic pathways, increase resistance to oxidative stress, and remodel their photosynthetic machinery [10,14,15].

When faced with episodic N inputs, diatoms have evolved rapid nitrate uptake and storage strategies [16,17] and are able to efficiently adjust intracellular N turnover, C metabolism, and energy balance to promote and optimize growth in the face of constantly fluctuating N substrate availability [18]. When N is not available, diatoms respond to N stress by diverting central carbon metabolism to generate lipids which store energy in the absence of growth and result in elevated C:N ratios [19,20,21]. Additionally, N stress results in decreased growth rate, photosynthesis and protein synthesis, while triggering oxidative stress responses [20,21].

Cellular responses to various nutrient limitation conditions are multifaceted and likely occur across different levels of cellular regulation (e.g., transcriptional vs. post-transcriptional vs. post-translational). While previous studies have focused on identifying and characterizing these responses through transcriptomics and proteomics, intracellular signaling mechanisms and pathways that trigger these cellular responses remain unclear. Phosphorylation-based signaling is one such way to control signal transduction between metabolomic pathways [22]. Phosphorylation, a ubiquitous form of post-translational modification, can activate and deactivate proteins. Current estimates suggest that the fraction of proteins regulated by phosphorylation range between 10 to 60%, depending on organism [23,24,25,26,27]. In commonly studied model eukaryotic organisms such as the yeast, *Saccharomyces cerevisiae*, and the vascular plant, *Arabidopsis thaliana*, ~44% and ~18% of the proteome has been shown to be subject to regulation by phosphorylated respectively [27,28]. In the unicellular photosynthetic eukaryotic green algae, *Chlamydomonas reinhardtii*, 23% of the proteome was found to be subject to phosphorylation [29].

To date, only one global phosphoproteome study has been conducted in any diatom. In the emerging model pennate diatom *Phaeodactylum tricornutum* [30] proteins observed to be phosphorylated during high light, and in response to Fe and N starvation were reported. Our study aims to develop a more comprehensive and condition specific phosphoproteome for *P. tricornutum*. We focus on the changes in proportions of phosphorylated proteins across diel cycles under differing Fe levels and over a time following shifting N availability by comparing the phosphoproteome and proteome. This phosphorylation dataset expands on prior transcriptomic studies by Smith et al. (2016 and 2019) under diel and varying Fe and N conditions [15,18]. Analyses of phosphorylation changes in the context of previously published physiological, transcriptomic, and proteomic studies provides a new perspective on the cellular response of diatoms to shifting environmental conditions. Regulatory signals controlled by phosphorylation in the model diatom, *P. tricornutum*, highlight a fundamental step in how diatoms sense changes in nutrient availability in their external environment which ultimately affects the growth and decline of populations. This research provides new insights by using the phosphoproteome as a tool to underscore proteins, as well as associated metabolic and regulatory mechanisms, that are important for nutrient sensing in diatoms.

## 2. Materials and Methods

### 2.1. Growth Conditions and Sampling

The proteome and phosphoproteome samples analyzed in this study were taken during previous experiments that investigated the cellular response to iron levels and diel cycling [15] and to short-term variation in nitrogen levels [18]. Detailed experimental designs and physiological data for these studies are publicly available [15,18] but are summarized briefly here to provide essential context to interpret the new results. For the iron experiments (referred to here as “Fe diel”), described in Smith et al. [15] cultures were grown in 20 L semi continuous batch cultures over a 12 h light:dark cycle (320 μEm^−2^ s^−1^) at 18 °C. Replicate experiments were conducted sequentially in modified f/2 artificial seawater medium in June and July [15]. Cultures were maintained in steady state (growth rates changed by less than 10%) at high (400 pM), medium (40 pM) and low (20 pM) Fe’ levels and sampled every four hours. Three samples were taken during the light period: 10 AM, 2 PM, 6 PM (lights on at 9 AM), and three during the dark period: 10 PM, 2 AM, 6 AM (lights off 9 PM) at each [Fe]. Fe diel physiological observations and transcriptomic analysis are further described in Smith et al. [15]. For statistical analyses, samples were binned by time point or by Fe level as indicated below (Section 2.4. Data Analysis).

To evaluate changes in the phosphoproteome and proteome in response to variable N, samples were taken during a short time course variable N experiment (referred to here as “Nshort”) that has recently been analyzed in depth at the transcriptome level [18]. In this experiment, *P. tricornutum* batch culture was grown in artificial seawater medium with f/2 nutrients (with 880 μM NH_4_ as the sole nitrogen source), trace metals, and vitamins, stirred and bubbled with air under 14:10 light:dark (150 μEm^−2^ s^−1^) at 18 °C. At mid-exponential phase, cells were split into four 800 mL treatments with (1) no nitrogen (N−), or [300 μM] of (2) ammonium, (3) nitrite, or (4) nitrate at a cell density of ~7.5 × 10^5^ cells mL^−1^. Treatments were then sampled at t = 15 min, 45 min, and 18 h in duplicate for transcriptome analysis [18] and in single replicate for proteome and phosphoproteome analysis (this study). Nutrient data collected at the 18 h timepoint show that nitrogen has been depleted similar to previous studies [31,32,33]. However, growth was detected (~1.2 doublings d^−1^) over this 18 h period (including in the N- treatment) indicating cultures had not yet become growth-limited or N-starved by the end of the experiment and cells were likely in early stages of sensing reductions in N availability (Table S1). This is corroborated by observations of the transcriptome from this experiment whereby all N source treatments exhibited hallmark transcripts of N limitation by 18 h [18], also seen in previous N-limitation studies [34,35]. While subtle differences were noted in the transcriptome of from different N source treatments, the dominant driver of changes in the transcriptome was overall nitrogen status (i.e., replete at t = 15 min, 45 min and deplete at 18 h) regardless of N source (Figure S1) [18]. Therefore, we treated individual N source time courses (ammonium, nitrite, and nitrate) as biological replicates to overcome statistical issues with analyzing data from single replicates. These samples are not true biological replicates and treating them as such means the ability to resolve the differences in proteome and phosphoproteome as a function of N source is lost, however this approach is justifiable since it robustly captures patterns that are driven by overall N status.

### 2.2. Phosphopeptide Extraction and Analysis

Protein samples taken for each experiment were digested, labeled, and analyzed with iTRAQ-based mass spectrometry (MS) at the Pacific Northwest National Laboratory as described in Smith et al. [18]. Samples were subjected to immobilized metal affinity chromatography (IMAC) for phosphopeptide enrichment. Briefly, magnetic Fe^3+^-NTA-agarose beads were prepared using Ni-NTA-agarose beads [36]. Peptides were reconstituted in 500 µL IMAC binding/wash buffer (80% MeCN, 0.1% TFA) and incubated for 30 min with 125 µL of the 5% pre-conditioned bead suspension. After incubation, the beads were washed 4 times each with 500 µL of wash buffer. Phosphorylated peptides were eluted from the beads using 125.0 µL of 1:1 acetonitrile/2.5% ammonia in 2 mM phosphate buffer (pH 8). Samples were acidified to pH ~3.5 and concentrated to 5–10 µL and subsequently reconstituted to 30 µL with 0.1% TFA for LC-MS/MS analysis.

### 2.3. Phosphopeptide Identification, Quantification and Manual Curation

Proteome and phosphoproteome peptides were identified using SEQUEST [37] as described in Smith et al. [18]. Peptide sequences were determined by MS-GF+ and phosphorylation sites (p-sites) on each peptide were determined by Ascore [38,39]. The median ratios of all non-phosphorylated peptides from the proteome study were used to normalize the ratios of all phosphorylated peptides. The MS proteomics data have been deposited to the ProteomeXchange Consortium. Raw data for Fe diel proteome, Fe diel phosphoproteome and Nshort phosphoproteome (PXD015059) and Nshort proteome (PXD015061) is available on PRoteomics IDEntifications database (PRIDE).

Data was further manually curated (Table S2) according to the following criteria. Since hydroxyproline and oxidized methionine containing peptide sequences were detected distinctly, these peptides were analyzed separately even if phosphorylation was seen on the same residue. Additionally, if multiple p-sites were detected on the same peptide in the same sample, it is likely that this peptide is commonly phosphorylated at multiple locations. Thus, these peptides were also separated and analyzed individually. If multiple p-sites were detected on the same peptide in different samples, we assumed that Ascore predictions were ambiguous and data was collapsed into a single peptide. If the same p-site was predicted on multiple peptides due to miscleavages, the full tryptic peptide with the strongest signal (data across all iTRAQ runs) was used for analysis.

### 2.4. Data Analysis

**Figure 1:**
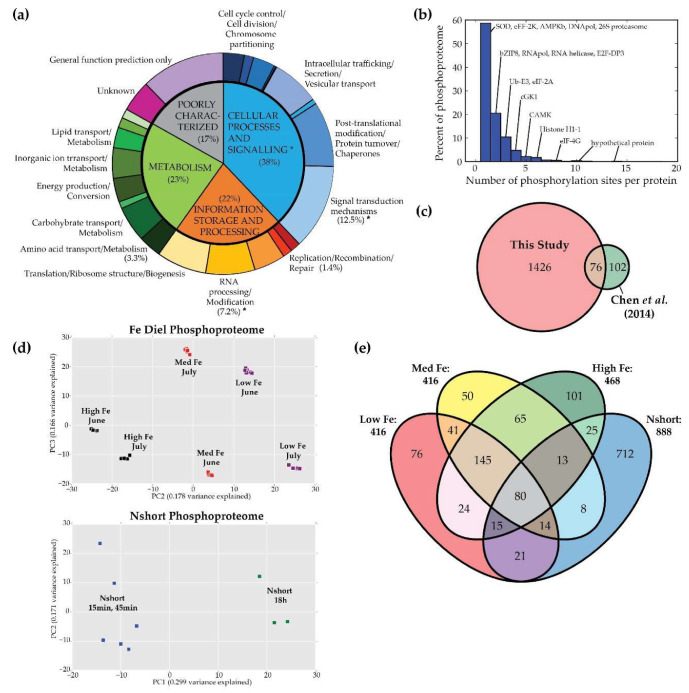
Summary of the phosphoproteome of *P. tricornutum*. (**a**) Proportions of detected phosphoproteins in each KOG class (* indicate significantly enriched groups, ^ indicate significantly depleted groups). Proteins related to signal transduction mechanisms and RNA processing/modification were significantly enriched for phosphorylation. (**b**) The majority of phosphoproteins (76%) had one or two phosphorylation sites. Highly phosphorylated phosphoproteins (>5 p-sites) was 5.6% of the total phosphoproteins. (**c**) This study detected 2759 p-sites on 1502 proteins, most of which were not previously detected by Chen et al. [30] in *P. tricornutum*. (**d**) PCA shows that phosphorylation in *P. tricornutum* is affected by nutrient availability and culturing environment. (**e**) Unique phosphopeptides detected during low (*n* = 76), medium (*n* = 50) and high (*n* = 101) Fe treatments or in Nshort experiments (*n* = 712) are likely candidates controlling cellular response and environmental sensing. Constitutively phosphorylated phosphoproteins (*n* = 80) are likely required for core cellular functions.

Phosphopeptides detected were mapped using Python 2.7.6 (Bio, SeqIO) to each protein to determine phosphorylation loci and number of p-sites per protein. This was then compared to the p-sites detected by Chen et al. [30]. Phosphopeptides and proteins were assigned annotations according to Kyoto Encyclopedia of Genes and Genomes (KEGG), Pfam and EuKaryotic Orthologous Groups (KOG) classes and subcellular localization was predicted based on methods described in Smith et al. [15,18]. “HYPGEOM.DIST” function in Excel 2016 was used to calculate hypergeometric enrichment *p*-values, based on the probability density function, between phosphoproteome and proteome to determine significantly enriched or depleted KOG groups and classes for phosphorylation (*p* < 0.001 was considered significant, Figure 1a). Principal component analysis (PCA) for phosphoproteome experiments was calculated using Python (sklearn.decomposition, PCA).

We first coded patterns in phosphopeptide detection across Fe diel and Nshort experiments (Table S1). Each phosphopeptide and protein was assigned a code to four positions corresponding to detection in the (1) low Fe time course, (2) med Fe time course, (3) high Fe time course, and (4) Nshort time course. A “1” indicates the phosphopeptide was detected in at least one sample from the condition, while “0” indicates it was not detected in any samples. These codes were then used to parse data into detection categories of biological significance that were analyzed further. Phosphopeptides detected at low Fe (LowFe-P; pattern = p1X0X, X = 1 or 0 permitted but not required, Table S4), at all Fe’ levels (candidates for diel-drive conditional phosphorylation, Diel-P; pattern = p111X, Table S5), and during Nshort (Nshort-P; pattern = pXXX1, Table S6). For the Diel-P subset (Table S5), log2 peptide abundances for Fe diel June and July replicates were averaged and hierarchically clustered using Morpheus (https://software.broadinstitute.org/morpheus), identifying 5 diel clusters. For the Nshort-P subset (Table S6), log2 fold changes (log2FC) were calculated between N replete (t = 15 min, 45 min) and early N deplete (t = 18 h) timepoints. Only replicates that had similar log2FC (standard deviation <25%) were analyzed in order to isolate the effect of N availability and reduce the effect of N source on the analysis. This filtered subset was then hierarchically clustered using Morpheus and 3 N clusters were identified. Phosphopeptides with a log2FC greater than 1 were considered significant.

Since phosphopeptides were enriched during sample preparation, their abundance cannot be quantitatively normalized to total protein abundance, presenting a challenge for determining conditional phosphorylation (in response to changing nutrient conditions for example). To overcome this and identify conditionally phosphorylated proteins, we compared patterns of phosphopeptide detection against the patterns of their respective proteins by calculating Pearson correlation coefficient (PCC). However, since the dataset was not robust enough to produce a normal distribution, PCC values could not be used as a cut off for analysis purposes and were instead only used to broadly indicate “anti-correlated” (PCC < −0.7) or “uncorrelated” (−0.2 < PCC < 0.2) phosphoproteins and proteins.

## 3. Results and Discussion

### 3.1. Overview

In this study, we compare phosphopeptide abundance to transcript expression and protein abundance data obtained at the same timepoint to determine phosphorylation changes and its possible impact on protein activity in two experiments, Fe diel and Nshort. Of the ~12,000 predicted nuclear-encoded proteins in the *P. tricornutum* genome, ~7700 proteins were detected in this study, which is more than double the number identified in previous *P. tricornutum* studies [20,21,40,41]. We detected 3059 unique phosphopeptides on 1502 proteins (Table S1). Phosphorylated proteins were found across diverse categories of cellular function but were enriched in categories with putative roles in signal transduction mechanisms (*p* = 1.9^−11^) and transcription (*p* = 2.9^−5^) relative to the total proteome (Figure 1a). Amino acid transport and metabolism (*p* = 5.9^−4^), and replication recombination and repair (*p* = 6.6^−4^) were proportionally depleted for phosphorylation. Phosphorylation on serine residues was most common (81%), followed by threonine (16%) and tyrosine (3%). The proportion of phosphoproteins with one and two p-sites were 55% and 21% respectively, and proteins with greater than five p-sites consisted of 5.6% of the detected phosphoproteins (Figure 1b). These proportions are similar to previously reported values in *A. thaliana* [42]. Of the annotated proteins that were highly phosphorylated, we found that 53% were involved in cellular processes and signaling, further highlighting the importance of phosphorylation in regulation of signaling cascades. However, the number of p-sites per protein does not appear to be correlated to protein function. For example, eukaryotic translation initiation factors and kinases have a large variability in the number of p-sites per protein, ranging from one to eight.

Since miscleavages and multiple p-sites were detected for each phosphopeptide, the 3059 phosphopeptides detected were mapped to the proteome identifying 2759 p-sites on 1502 proteins, the majority of which were not previously detected in *P. tricornutum* (Figure 1c, Table S3). Despite differences in experimental conditions and sample processing methods we detected ~35% of the phosphoproteins previously identified by Chen et al. [30]. Notable examples include PPDK (Phatr3_J21988) and EF-3A (Phatr3_EG02323), which were detected in all samples of this study and MARK3 (Phatr3_J8773) which is phosphorylated during low Fe and low N conditions.

To broadly examine variation within and between experimental conditions, principal component analysis (PCA) was performed (Figure 1d). Samples separated along principal component 2 (PC2) by treatment (low, medium, high Fe). However, batch effects were also a major variable and it appears that phosphoproteomics could be more sensitive to cellular and experimental conditions than transcriptomic data [15,18].

### 3.2. Conditional Phosphorylation under Fe Limitation, Across Diel Light Cycling, and under N Stress

**Figure 2:**
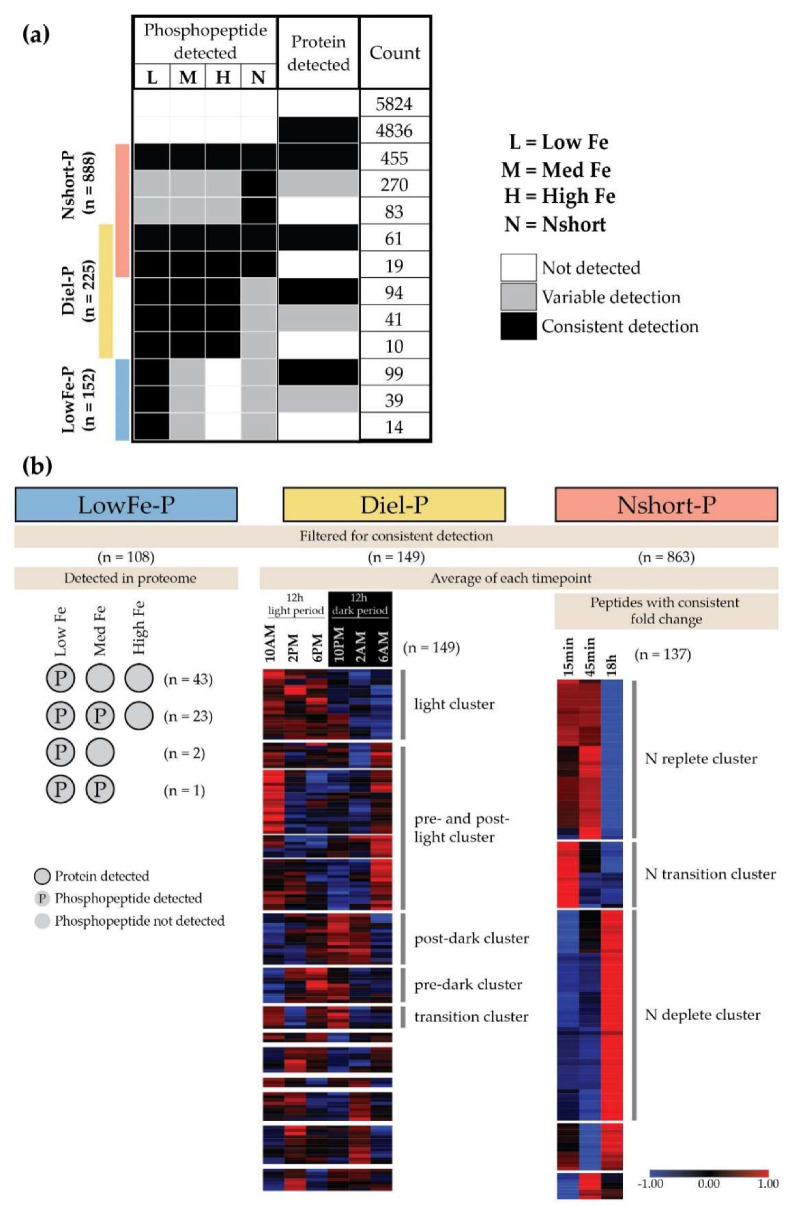
Division of phosphoproteome into conditional subsets. (**a**) Patterns of conditional phosphorylation compared to total protein expression as a response of *P. tricornutum* to low Fe (LowFe-P), diel light cycling (Diel-P), and N availability (Nshort-P) consisted of 10%, 15% and 59% of the phosphoproteome respectively. (**b**) Breakdown of LowFe-P subset by pattern. Diel-P Fe treatments were averaged by timepoint to highlight the phosphorylation changes due to the diel cycle. Similarly, to illustrate the response to cellular N status, Nshort-P nitrogen treatments were averaged and log2FC was calculated between timepoints. Normalized phosphopeptide abundances were hierarchically clustered.

Proteins that are conditionally phosphorylated as a function of nutrient status are good candidates for regulatory components of the cellular response to varying conditions. Of the 3059 phosphopeptides detected in this study, 76, 50 and 101 phosphopeptides were uniquely detected at low, medium and high Fe treatments respectively, while 712 phosphopeptides were detected in the Nshort experiments (Figure 1e). Patterns of protein and phosphopeptide expression were binned according to experiment and treatment into 3 categories, LowFe-P, Diel-P and Nshort-P (Figure 2). Phosphopeptides which were consistently absent during high Fe were classified as LowFe-P, phosphopeptides which were phosphorylated under all Fe treatments were classified as Diel-P and those which were detected under all Nshort treatments were classified as Nshort-P (Figure 2). Of these, phosphoproteins also detected in the proteome made up 10%, 15% and 59% of the phosphoproteome for LowFe-P (Table S3), Diel-P (Table S4) and Nshort-P (Table S5) subsets respectively (Figure 2).

### 3.3. Low Fe Phosphorylation

Of the LowFe-P consistently detected in the proteome (*n* = 108), we found 12 phosphoproteins, including flavodoxin (Phatr3_J23658), E3 ubiquitin protein ligase (E3; Phatr3_J12887), ferrocytochrome oxygenase (HO-1; Phatr3_J12588) and 9 hypothetical proteins, that were previously characterized as transcriptionally responsive to low Fe [15]. We hypothesize that flavodoxin, E3 and HO-1 are active when phosphorylated as their phosphorylation is Fe dependent despite consistent protein expression under all Fe treatments. Flavodoxin is needed as a substitute for ferredoxin in photosynthetic electron transfer chains [43], E3 assists in protein and mRNA turnover [44], and HO-1 is stress response protein responsible for the breakdown of heme [45]. Besides these 12 LowFe-P phosphoproteins, gene products that are transcriptionally sensitive to low Fe (20 pM) do not appear to also be regulated by phosphorylation. This suggests that transcriptional and post-translational regulation is non-redundant, and that important aspects of the low Fe response of diatoms also need to be considered from a post-translational regulatory perspective. Besides the phosphoproteins identified in the LowFe-P subset, we note that some iron responsive phosphoproteins, such as ISIP2A phytotransferrin (pTF; Phatr3_J54465), were excluded from the LowFe-P subset due to phosphoproteome data sparseness despite indications that they are also Fe responsive.

Transcript expression of central carbon metabolism genes were relatively unaffected by low Fe [15]. However, carbon metabolism phosphoproteins, NAD-glutamate dehydrogenase (GDH2; Phatr3_J45239), 2-oxoglutarate dehydrogenase (2OGDH; Phatr3_J29016), phosphoglycerate mutase (Phatr3_J45200), and mannose-6-phosphate isomerase (Phatr3_J10693) were LowFe-P. Although regulation by phosphorylation has not been studied for these proteins at low Fe, our results suggest that glycolysis, the citric acid cycle (TCA) and N metabolism may be regulated by phosphorylation in response to low Fe conditions. Specifically, the phosphorylation of GDH2 and 2OGDH only under low Fe, possibly to regulate the alpha ketoglutarate pool, emphasizes the tight coupling of C and N metabolism and associated decrease in C:N ratios under low Fe in diatoms [46]. Furthermore, C degradation proteins, catalase (Phatr3_J22418) and glycosyl hydrolase (Phatr3_J1372), were also LowFe-P, indicating these proteins are likely activated by phosphorylation and involved in reallocation of C under low Fe.

LowFe-P proteins involved in transcription and translation may also control gene expression and protein abundance under low Fe. Phosphorylation of the FACT complex subunit SSRP1 (Pt_SSRP1; Phatr3_EG02355) and CCR-associated factor 1 (CAF1; Phatr3_J9576) could have implications for transcriptional control under low Fe. The FACT complex binds to DNA, destabilizing one histone dimer from the nucleosome, facilitating transcription. In humans, when SSRP1 of the FACT complex is phosphorylated on Ser-510 by casein kinase 2, DNA binding is inhibited [47]. Both human SSRP1 and Pt_SSRP1 are phosphorylated on serine residues and protein alignments show that the p-sites are within six residues of each other. Thus, it is likely that in *P. tricornutum*, phosphorylation of Pt_SSRP1 at low Fe also inhibits DNA binding, resulting in reduced transcription.

Phosphorylation of ribosomal proteins (RP) S3 and L24 (Phatr3_J17545, Phatr3_J19152 respectively), may influence translation or trigger signaling at low Fe. Ribosome phosphorylation results in conformational change and reduced protein synthesis [48]. However, in eukaryotes only the phosphorylation of RPS6 has been extensively studied. It has been linked to mTOR signaling pathways involved in regulation of translation [49,50]. Phosphorylation of RPS6 may also promote selective translation and assist in the formation of polysomes [51,52]. In addition to RPs, tRNAs are also an important part of translation and could be modified as a response to stress [53]. The elongator complex protein 1 (Pt_ELP1; Phatr3_J43627) is in the LowFe-P group and likely plays a role in regulating translation. ELP1 has a wide variety of functions in many organisms, including transcriptional-silencing and nucleosome assembly [54]. In yeast, phosphorylation of ELP1 on 4 serines at the C-terminus has been hypothesized to promote wobble uridine tRNA modification, favoring the translation of mRNA particularly reliant on ELP1 elongator function [55]. Although only one p-site was found for Pt_ELP and it was a pThr instead of a pSer, protein alignments indicate that Pt_ELP pThr is at a similar location to the yeast homolog. Furthermore, the yeast wobble uridine tRNA modification pathway is well conserved between yeast and plants [56]. Taken together, phosphorylation of Pt_ELP could indicate preferential translation of certain proteins under Fe limitation in diatoms.

### 3.4. Diel Profiles of C and N Metabolism Phosphoproteins

**Figure 3:**
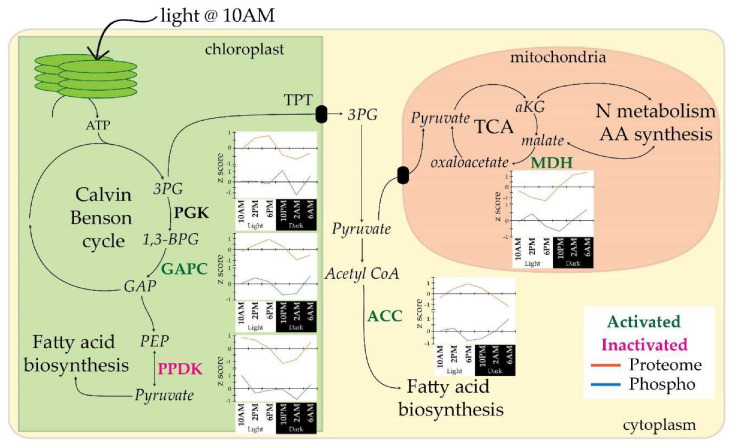
Phosphorylation of carbon metabolism gene products suggests a role for phospho-regulation in directing carbon into fatty acid biosynthesis. Phosphoproteins were identified to be inactivated at 6 AM but reactivated at 10 AM (green, bold), inactivated at both 6 AM and 10 AM (pink, bold) or undetermined (black, bold). Diel profiles normalized across the time course (z score) indicate phospho-regulation as the change in phosphorylation (blue line) is not proportional to the change in total protein abundance (orange line) between timepoints. Chloroplast phosphoglycerate kinase (PGK; Phatr3_J29157); chloroplast glyceraldehyde 3-phosphate dehydrogenase (GAPC; Phatr3_J22122); chloroplast pyruvate orthophosphate dikinase (PPDK; Phatr3_J21988); cytoplasmic acetyl-CoA carboxylase (ACC; Phatr3_J55209), mitochondrial NAD-dependent malate dehydrogenase (MDH; Phatr3_J42398). Refer to Table S7 for a summary of the possible effect of phosphorylation on these phosphoproteins.

Light induced phosphorylation has been observed in bacteria [57], plants [58,59] and mammals [60]. In photosynthetic organisms, light availability leads to increased ATP pools, which kinases can use to phosphorylate proteins. In *A. thaliana*, a phytochrome-interacting transcription factor is phosphorylated in response to red and far-red light resulting in its degradation to promote photomorphogenesis [58]. In *Spinacea oleracea*, the plant-specific thylakoid soluble phosphoprotein was phosphorylated during the light period and was ultimately hypothesized to control gene expression of photosynthesis genes through redox-mediated signal cascade [59]. Hierarchical clustering of Diel-P (*n* = 149) highlighted 5 clusters of phosphoproteins that changed over the 12h light:dark diel cycle (Figure 2b). In the light clusters, most phosphoproteins appear to play a role in C and N metabolism. Phosphorylation profiles of C metabolism genes in the pre- and post-light cluster appear to direct C flux towards fatty acid and amino acid biosynthesis. Chloroplast phosphoglycerate kinase (Pt_PGK; Phatr3_J29157, PCC = uncorrelated), chloroplast glyceraldehyde 3-phosphate dehydrogenase (GAPC; Phatr3_J22122, PCC = uncorrelated), and cytoplasmic acetyl-CoA carboxylase (Pt_ACC; Phatr3_J55209, PCC = anti-correlated) are inactivated/phosphorylated pre-light (6 AM) and activated/dephosphorylated post-light (10 AM), suggesting increased C flux through the Calvin Benson cycle and enhanced shuttling of C skeletons towards fatty acid biosynthesis (Figure 3). Phospho-regulation of PGK has not been previously reported although p-sites have been documented [61]. In both *A. thaliana* PGK and Pt_PGK, phosphoserines were identified, Ser-81 [61] and Ser-83 respectively. Phosphorylation of Calvin Benson cycle GAPC has also not been previously reported but phosphorylation of the cytosolic glyceraldehyde 3-phosphate dehydrogenase homolog, *A. thaliana* GAPN, has been previously investigated. Phosphorylation of *A. thaliana* GAPN results in a lower Vmax, increased sensitivity to inhibition by ATP and disruption of GAPN binding to 14-3-3 signaling proteins, affecting both protein function and signaling [62]. Thus, phosphorylation of GAPC in *P. tricornutum* may be of importance. ACC controls C flux into lipid metabolism and appears to have multiple p-sites. The phosphorylation of mouse ACC1 at Ser-79 and mouse ACC2 at Ser-212 by AMP–activated protein kinase has been shown to inhibit activity [63,64]. Pt_ACC p-site Ser-1168, detected here, aligns with observed p-sites in mouse ACC2 (pSer-1332) [65] and is 2 residues from pSer-1200 of rat ACC1 [66]. While phosphorylation of these pSer residues has been observed, regulatory significance of these p-sites has not been determined. Finally, chloroplast pyruvate orthophosphate dikinase (Pt_PPDK; Phatr3_J21988), was also observed to be phosphorylated pre- and post-light, 6 AM and 10 AM respectively. The p-site detected in Pt_PPDK aligned with Thr-527 in *Zea mays* [67,68]. Phosphorylation by PPDK regulatory protein at pThr inactivates the PPDK, favoring pyruvate formation from phosphoenolpyruvate (PEP) [67,68]. With the accumulation of pyruvate in the chloroplast, phosphorylation/inactivation of PPDK further supports our hypothesis that phospho-regulation in diatoms serves a general purpose of promoting the shuttling C towards fatty acid biosynthesis.

Mitochondrial NAD-dependent malate dehydrogenase (Pt_MDH; Phatr3_J42398) of the TCA cycle is also phosphorylated pre-light (6AM) and dephosphorylated post-light (10AM). In *S. cerevisiae*, phosphorylation of MDH2 at Ser-12 results in inactivation and possible degradation [69]. Although phosphorylation of Pt_MDH is not at the same loci (p-site aligned to Sc_MDH2 Ser-4 instead), both p-sites are in close proximity at the N terminus. Additionally, both Ser-4 and Ser-12 of *S. cerevisiae* MDH2 are conserved in Pt_MDH. Thus, considering the phosphorylation profile, sequence conservation and previous phosphorylation studies in yeast, we hypothesize Pt_MDH is inactivated by phosphorylation pre-light and is only active/dephosphorylated post-light. Since activity of MDH affects the malate pool and hence, the alpha ketoglutarate reservoir of the cell, it represents an important link between C and N metabolism. Furthermore, the product of MDH, oxaloacetate can be converted back into PEP.

To further emphasize the potential impact of phosphorylation on cellular C allocation, we highlight that three of the five phosphoproteins discussed in the pre- and post-light cluster (Pt_ACC, Pt_PPDK, Pt_MDH) have key roles in the PEP-pyruvate-oxaloacetate node. The PEP-pyruvate-oxaloacetate node is responsible for partitioning and directing C flux to catabolic, anabolic, and energy production pathways [70]. Tight phospho-regulation of these three C metabolite pools within and across intracellular compartments might enable efficient modulation of C flux in response to shifting conditions. Our observations show that phosphorylation controls the activity of key proteins at the PEP-pyruvate-oxaloacetate node and thus, phosphorylation may be a fundamental method for intracellular control of C flux.

C and N flux are also tightly coupled within the cell and N assimilation genes, such as nitrate and nitrite transporters, nitrate reductase and glutamine synthetase, were shown to be transcriptionally upregulated during the day [15]. Although, only 3 N metabolism phosphoproteins were found in the light cluster (Figure 2; Figure S2) and information on the phospho-regulation is sparse, we hypothesize that phosphorylation inhibits activity of 2 of the 3 proteins; plasma membrane localized urea symporter (DUR3; Phatr3_J20424, PCC = uncorrelated) and cytoplasmic glutamine-dependent carbamoyl phosphate synthetase (pgCPS; Phatr3_EG01947, PCC = anti-correlated). pgCPS was phosphorylated on Ser-970, which was not a conserved residue when aligned with the eukaryotic orthologs *A. thaliana* CPSB or human CAD. It was interesting to note that pgCPS transcript expression was upregulated immediately after light transitions (10 AM/10 PM) while protein accumulates after the dark [71] when phosphorylation is low. pgCPS is part of the pyrimidine biosynthesis pathway and we hypothesize that pgCPS is active in the dark indicating that its phosphorylation is inhibitory. Plasma membrane localized DUR3 was phosphorylated on Ser-70, which is a conserved serine residue in *A. thaliana* DUR3 (53.5% identity). Although no phosphorylation sites are currently known for this protein, the transcript is upregulated at night and is preferentially phosphorylated in the day despite protein abundance remaining relatively stable. Our hypothesis is that phosphorylation inhibits urea transport by DUR3 during the day when urease expression is downregulated and the catabolic component of urea cycle activity is low in diatoms [15,71].

The other phosphoprotein with a potentially direct role in N metabolism is a vacuolar sorting-associated protein (VPS; Phatr3_EG02146, PCC = uncorrelated). VPS was found to be phosphorylated on Ser-193, at an un-conserved region upstream of the Vps35 functional domain when aligned to *A. thaliana* VPS35 (29.6% identity). *A. thaliana* VPS has been shown to interact with aquaporins, localize to the vacuolar membrane and is a candidate for facilitating N transport across the vacuolar membrane [72,73]. Phosphorylation of VPS peaked post-light (10 AM) and steadily decreased thereafter while transcriptomic and proteome profiles do not show a distinguishable diel expression pattern. Additionally, PCC indicates that VPS phosphorylation and protein abundance are uncorrelated (Figure S2). We suggest VPS as a candidate for further study but cannot currently hypothesize on the impact of phosphorylation on this protein.

### 3.5. Translation Is Impacted by Coordinated Phosphorylation over the Diel Cycle

Over the course of the 12 h light:dark diel cycle, 6 samples were collected (light 1 h, 5 h, 9 h and dark 1 h, 5 h, 9 h) and phosphorylation of different translation related proteins oscillated, possibly affecting translation rates and ribosomal binding to mRNA. Two such phosphoproteins were small ribosomal subunit 40 S9 (Pt_S9; Phatr3_J6847, pre- and post-light cluster) and 60S acidic ribosomal protein P0 (P0; Phatr3_J30660, PCC = uncorrelated, pre-dark cluster). Protein abundance and phosphorylation of Pt_S9 was well correlated (Figure S2) and relatively stable throughout the day with a dip at 2 AM and a peak at 6 AM. The decrease at 2 AM may be related to the observed dark period cell division, predicted from transcriptomic data and counts of dividing cells [15], while the increase at 6 AM could be in preparation for increased demand for translation during the light period, as observed in *A. thaliana* [74,75]. As such, phosphorylation of Pt_S9 can be hypothesized to be important for ribosomes. However, the p-site Ser-135 of Pt_S9 found did not align with known p-sites, Ser-153 and Ser-163, of human S9 homologs [76]. In contrast, protein abundance and phosphorylation profiles were uncorrelated for P0 (Figure S2). P0 is essential for growth and is part of the ribosomal stalk of the 60S ribosomal large subunit where it binds to P1 and P2 dimers and possibly interacts with elongation factors [77]. P0 protein abundance was highest at 2 AM with a secondary peak at 2 PM while phosphorylation peaked at pre-dark (6 PM). The p-site of P0, Ser-115, was also not conserved in the human homolog. However, research on the human P0 homolog shows that multiple kinases are responsible for P0 phosphorylation and it is believed that, although phosphorylation does not affect ribosome assembly, ribosomal activity and regulation may be affected by phosphorylation [78]. Thus, we hypothesize that pre-dark may be an important time for translation in the cell to synthesize proteins required in preparation for the dark such as transporters and cell division proteins [15].

Phosphoproteins detected that may affect translation elongation include soluble elongation factor 3A (Pt_EF3A; Phatr3_EG02323, PCC = uncorrelated) and eukaryotic elongation factor 2 kinase (Pt_eEF2K; Phatr3_EG02345, PCC = uncorrelated), which were both part of the pre- and post-light cluster. EF3A interacts with the small ribosomal subunit to control translational accuracy in yeast in a mechanism that is similar to its bacterial homolog [79,80]. Yeast EF3A homolog (YEF3P) has a 43% identity to Pt_EF3A and p-site Ser-2244 aligns with pSer-1040 of YEF3P. Notably, dephosphorylation of Pt_EF3A occurs at 2 PM and 2 AM, which are the same timepoints as P0 protein peaks (Figure S2). Assuming that ribosome abundance and hence, translation, peaks at 2 PM and 2 AM, dephosphorylation of Pt_EF3A may impact translation accuracy by modifying the fidelity of the tRNA:ribosome interaction [80]. eEF2K may be responsible for EF3A phosphorylation. Human eEF2K has been shown to phosphorylate another soluble elongation factor, eEF2 [81]. When eEF2K is inhibited, translation progresses; and when eEF2K is active, it phosphorylates eEF2, preventing it from binding to ribosomes and halting translation [81]. The function of eEF2K is not disputed. However, its interaction with kinases and putative response to phosphorylation is complex. Human eEF2K has been shown to be part of the mTOR signaling pathway and is phosphorylated by multiple kinases with differing effects on eEF2K function, including AMPK which activates and S6K which is inhibitory [82,83]. Despite the many published p-sites on the human homolog, Pt_eEF2K p-site Ser-1214 is not conserved between *P. tricornutum* and human eEF2K. While it is difficult to extrapolate conclusions about the relationship between phosphorylation of eEF2K and its activity, it is noted that the phosphorylation profile of Pt_eEF2K is very similar to the protein abundance profile of Pt_EF3A (Figure S2). Taken together, our data suggest that Pt_eEF2K and Pt_EF3A likely interact, ultimately regulating translation elongation and translational accuracy.

In addition to ribosome abundance and translation elongation, translation initiation, which enables ribosomal binding to mRNA, is also essential. Two eukaryotic translation initiation factors, eIF4F (Phatr3_J44383, pre-dark cluster) and eIF4G3 (Phatr3_EG02122, PCC = uncorrelated, light transition cluster), were identified to have phosphorylation patterns in response to diel light cycling. eIF4F phosphorylation peaks during the day (2 PM to 6 PM) while eIF4G3 phosphorylation was highest post-dark (10PM) with a secondary peak post-light (10 AM). To date, eIF phosphorylation has not been extensively investigated. We found four p-sites for eIF4F and eight p-sites for eIF4G3 in our dataset. However, only phosphopeptide abundance of pSer-9 of eIF4F and pSer-574 of eIF4G3 seem to be affected by the diel cycle unlike the other p-sites detected on these eIFs. eIF4F and 3IF4G3 are both part of the “cap building complex” and are important for translation. Specifically, eIF4F allows for mRNA discrimination enabling variation in mRNA translation rates [84] and eIF4G is part of the eIF4F complex, providing docking sites for other eIFs [85]. eIF4G interacts with eIF4E or eIF4E binding proteins, either initiating or inhibiting translation respectively [85]. In cancer research, inhibition of eIF4F through the inhibition of eIF4E induces tumor cell death [84] and has been the subject of extensive research. Since mTOR phosphorylation has been connected to the function of eIF4G and eIF4F in humans [84,85], diel phosphorylation of both these eIFs may link to TOR signaling in diatoms.

### 3.6. Novel Phosphorylation of Fe Sensitive Proteins

Novel phosphorylation of two important iron-sensitive proteins, flavodoxin and phytotransferrin (pTF; PCC = uncorrelated), was detected in the phosphoproteome. Transcriptional levels for each of these gene products are known to increase in response to low Fe conditions (20 pM). mRNA expression levels for flavodoxin and pTF are commonly used as molecular markers of Fe stress in marine phytoplankton communities [86,87,88]. Chloroplast localized flavodoxin is phosphorylated at Tyr-28 just upstream of the functional domain. As stated previously, phosphorylation of this p-site is Fe dependent and likely activates flavodoxin. Recent focus on plasma membrane localized pTF in diatoms has shown that it is essential for high affinity inorganic Fe’ uptake [89,90,91] and responsive to the diel light cycle. Fe-uptake rates, transcript and protein abundance have also been shown to increase in the dark [15,92]. Total protein abundance of pTF in our dataset further illustrates that the diel pattern of pTF protein is significantly impacted by Fe, with a single protein peak at 6 AM at low Fe and a double peak under medium (40 pM) and high (400 pM) Fe treatments (Figure S3). Three p-sites were detected for pTF; Ser-26, Ser-525 and Thr-526. Two of these p-sites, Ser-26 and Thr-526, display phosphorylation profiles that appear to be affected by Fe. Phosphorylation at Ser-26 peaked at 6 PM and 6 AM at low and medium Fe while a different phosphorylation profile was observed at high Fe (Figure S3). Phosphorylation at Thr-526, on the other hand, was detected only under low Fe. P-sites for both flavodoxin and ISIP2A could be important for Fe sensing and utilization in the cell.

### 3.7. Phosphorylation in Response to Changes in Nitrogen

Two N replete timepoints (15 min and 45 min) and one early N deplete timepoint (18 h) were sampled and log2 fold change (log2FC) of phosphopeptide abundance was calculated to identify phosphoproteins that respond to fluctuating N (Table S5). Since some phosphorylation may be N source specific, only phosphopeptides that had a consistent log2FC (SD < 25%) across N treatments, which each manipulated total N availability, were considered, resulting in the Nshort-P subset.

**Figure 4:**
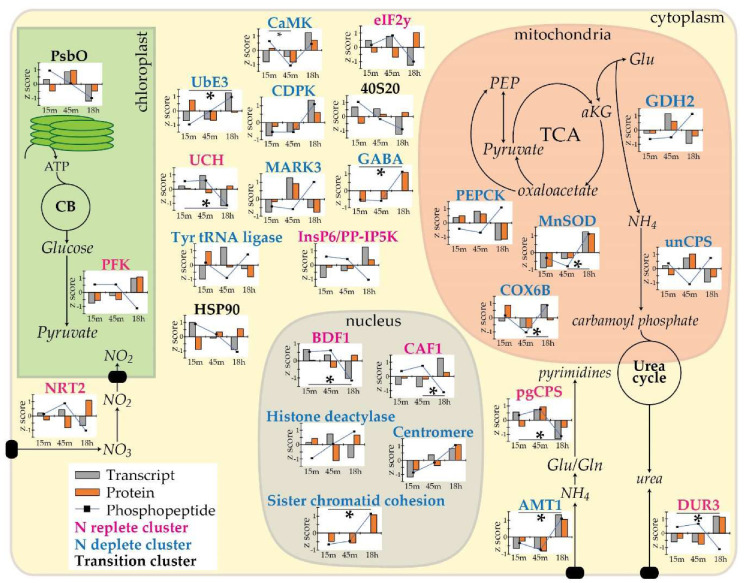
Phosphoproteins which are impacted by cellular nitrogen status. Transcripts (grey) [18], protein (orange) and phosphoprotein (blue line) levels are depicted. Phosphoproteins were classified into N replete (pink), N deplete (blue), or N transition (black) clusters by hierarchical clustering. Significant fold change (log2 FC > 1) in phosphopeptide abundance between timepoints are indicated by an asterisk on the graphs. Phosphofructokinase (PFK; Phatr3_EG02209), nitrate/nitrite transporter (NRT2; Phatr3_J2171), ubiquitin carboxyl-terminal hydrolase (UCH, Phatr3_J42729), ubiquitin E3 ligase (UbE3; Phatr3_EG01549), Tyr-tRNA ligase (Phatr3_EG02310), heat shock protein 90 (HSP90; Phatr3_J55230), Ca2+/calmodulin-dependent protein kinase (CaMK; Phatr3_EG02294), calcium dependent protein kinase (CDPK; Phatr3_J25067), serine/threonine kinase (MARK3; Phatr3_J8773), inositol hexakisphosphate/diphosphoinositol-pentakisphosphate kinase (InsP6/PP-IP5K; Phatr3_J46684), 40S small ribosomal subunit 20 (40S20, Phatr3_J51291), translation initiation factor eIF2 gamma (eIF2y; Phatr3_J42307), gamma-aminobutyric acid type B receptor (GABA; Phatr3_Jdraft1756), phosphoenolpyruvate carboxykinase (PEPCK; Phatr3_EG02232), NAD-glutamate dehydrogenase (GDH2; Phatr3_J45239), manganese superoxide dismutase (MnSOD; Phatr3_J42832), cytochrome C oxidase subunit 6b (COX6B; Phatr3_J11016), ammonium-dependent carbamoyl-phosphate synthetase (unCPS; Phatr3_J24195), transcription initiation factor TFIID subunit BDF1 (Phatr3_J44399), CCR-associated factor 1 (CAF1; Phatr3_J9576), histone deacetylase (Phatr3_J13057), centromere (Phatr3_J43019), sister chromatid cohesion protein (Phatr3_Jdraft1590), cytoplasmic glutamine-dependent carbamoyl phosphate synthetase (pgCPS; Phatr3_EG01947), ammonium transporter (AMT1; Phatr3_J27877), urea symporter (DUR3; Phatr3_J20424). Refer to Table S7 for a summary of the possible effect of phosphorylation on these phosphoproteins.

Based on hierarchical clustering of these 137 phosphoproteins, three clusters were apparent: N transition, N replete, and N deplete clusters (*n* = 17, 43 and 69 respectively; Figure 2). Phosphoproteins of interest in these clusters consisted of proteins that play key roles C and N metabolism, transcription and translation related proteins, and kinases. The predicted localization of these proteins and their role in metabolism, if known, is summarized in Figure 4.

N transporters and N metabolism proteins, especially mitochondrial proteins, were phosphorylated in response to changes in cellular N status. Phosphorylated transporters included plasma membrane urea symporter DUR3, ammonium transporter (AMT1; Phatr3_J27877), and nitrate/nitrite transporter (Pt_NRT2; Phatr3_J2171). Interestingly, the p-site of DUR3 was at Thr-69 instead of the diel responsive Ser-70. Transcript and protein profiles of DUR3 both increase during N limitation while phosphopeptide abundance of Thr-69 decreases significantly (log2FC = −1.04, Figure 4). This suggests that dephosphorylation of DUR3 at Thr-69 increases affinity or transport of urea into the cell as a response to N limitation. The opposite seems to hold true for AMT1. In our dataset, AMT1 is phosphorylated at 5 different p-sites, and only Ser-477 appears to be N responsive. Transcript, protein and phosphopeptide abundances increase under N limitation (Figure 4), suggesting phosphorylation either activates AMT1 (log2FC = 1.31) or that phosphopeptide levels increases proportionally with total protein abundance. Finally, phosphorylation of Pt_NRT2 at Ser-578 is also N responsive, with decreased phosphorylation during N deplete conditions despite high total protein abundance (Figure 4). Phosphorylation of nitrate transporters in higher plants and yeast have been shown to be involved in sensing and optimizing uptake of nitrate [93]. In *A. thaliana*, phosphorylation of NRT1 has a regulatory function while phosphoserines detected in homologous NRT2 proteins are not known to be regulatory [94]. In *Hansenula polymorpha*, yeast nitrate transporter (YNT1) is phosphorylated on Ser-246, by nitrogen permease reactivator 1, as a response to suboptimal nitrate levels [95]. This triggers accumulation of YNT1 on the plasma membrane, increasing the efficiency of nitrate transport into the cell [96]. However, Ser-246 of YNT1 is not conserved in *P. tricornutum* and Ser-578 of Pt-NRT2 is not conserved in *H. polymorpha*. Furthermore, despite protein abundance increasing during nitrogen depletion, Pt_NRT2 was dephosphorylated, which is opposite of YNT1. Phylogenetically, diatom NRT2s appear to be derived from the secondary exosymbiont and should function more similarly to yeast NRT2 [97]. Since transcript and protein of Pt_NRT2 are anti-correlated (Figure 4), we hypothesize that dephosphorylation may be the cause of transcript/protein decoupling and allow for accumulation of Pt_NRT2 in order to ultimately increase N scavenging under N deplete conditions.

In addition to transporters, C and N metabolism proteins affected by cellular N status include, phosphoenolpyruvate carboxykinase (Pt_PEPCK; Phatr3_EG02232), GDH2, ammonium-dependent ornithine urea cycle (OUC) type carbamoyl-phosphate synthetase (unCPS; Phatr3_J24195) and pgCPS. PEPCK is responsible for the conversion of oxaloacetate to PEP and in *Panicum maximum*, PEPCK phosphorylation decreases its affinity for its substrates; resulting in a 6-fold decrease in activity [98,99]. The predicted phosphorylated serine residue of *P. maximum* PEPCK [99] was not conserved in Pt_PEPCK, which was phosphorylated in the long unconserved N terminus at Ser-367. Pt_PEPCK transcript and protein levels were downregulated under N deplete conditions, while phosphopeptide abundance increased. Therefore, we hypothesize that Pt_PEPCK activity is not favored under conditions that promote low cellular N levels, and phosphorylation is inhibitory. We also hypothesize that phosphorylation results in decreased GDH2 activity. In the yeast, *Candida utili*, phosphorylation lowers activity of GDH by 10-fold [100]. The p-site responsible for this has not been determined. In *P. tricornutum*, GDH2 is phosphorylated at the same p-site (Ser-67) during both low Fe and low N conditions. Transcript and protein levels for GDH2 were downregulated during N deplete conditions (Figure 4), suggesting that GDH2 is not required for N reallocation and that phosphorylation and associated inhibition of GDH2 occurs in response to N limitation.

Phosphorylation of unCPS is anti-correlated while phosphorylation of pgCPS is relatively well correlated to respective transcript and protein levels (Figure 4). unCPS is localized to the mitochondria and drives the first committed step of the urea cycle, generating carbamoyl phosphate from ammonium, and is phosphorylated at Ser-209. Information on the phosphorylation of unCPS is sparse but Ser-209 is conserved in both pgCPS and human CPS proteins. Transcript, protein and phosphopeptide abundance of unCPS suggest phosphorylation and associated inhibition of unCPS in N response to limited conditions. On the other hand, phosphopeptide abundance of pgCPS is high during N replete conditions when transcript and protein levels for pgCPS are also high and decreases during N deplete conditions as transcript and protein abundance decrease (Figure 4). Drawing from our Fe diel observations above and the transcriptional response of pgCPS in *Thalassiosira pseudonana* to diel light cycling conditions [71], we maintain that phosphorylation of pgCPS at Ser-970 is inhibitory and suggest that pyrimidine is reduced during N deplete conditions. A known p-site of pgCPS human ortholog CAD is Thr-456. Although phosphorylation of Thr-456 by MAPK activates CAD, resulting in increased affinity for magnesium ions [101,102], this locus is not conserved in pgCPS in *P. tricornutum*. Hence, further supporting the hypothesis that pgCPS phosphorylation at Ser-970 inhibits protein activity. During N deplete conditions, although pgCPS transcript and protein levels are decreased, dephosphorylation may still allow for small amounts of pyrimidine biosynthesis.

### 3.8. Inhibition of Transcription and Translation as an Early Response to N Depletion

Translation is also affected by phosphorylation in response to changes in N status. We noted that phosphorylation of 40S small ribosomal subunit 20 (40S20, Phatr3_J51291) and translation initiation factor eIF2 gamma (Pt_eIF2y; Phatr3_J42307) correlates well with transcript abundance instead of protein abundance. Previous research has shown that phosphorylation of a protein could directly impact its transcript expression in a signaling feedback loop. For example, phosphorylation of HnoC in *Shewanella oneidensis* results in transcription derepression of HnoC [103]. Thus, 40S20 and Pt_eIF2y may have a similar regulatory feedback loop to drive increased transcriptional output for ribosomal and proteins required for translation. Alternatively, phosphorylation may also affect the function of the protein. eIF2 interacts with methionine initiator tRNA and binds to the 40S ribosomal subunit. Phosphorylation of Pt_eIF2y is unknown but detailed research has been conducted on the phospho-regulation of human eIF2 alpha (eIF2a). Phosphorylation of eIF2a can result from nutrient deficiency and can lead to overall down regulation of protein synthesis [104]. In yeast, phosphorylation of eIF2a results in increased affinity for eIF2B, trapping it as an inactive complex and preventing translation [104]. mTOR signaling is also affected by the phosphorylation state of eIF2 [105]. In the fungus *Neurospora crassa*, phosphorylation of eIF2a was found to be controlled by the circadian clock. It is phosphorylated during the day, leading to reduced translation during the day [106]. Although eIF2a and eIF2y are not identical and phospho-regulation may not be similar, it is possible that phosphorylation of eIF2y in *P. tricornutum* may impact protein synthesis and TOR signaling as a response to N depletion.

Finally, we identified two nucleotide binding proteins that had significant decreases in log2FC in phosphopeptide abundance between N replete and N deplete conditions, CAF1 and transcription initiation factor TFIID subunit BDF1 (Phatr3_J44399). In yeast and mammalian cells, the CCR4-CAF1-NOT complex associates with microRNAs, destabilizing them and resulting in translational repression of target mRNAs [107,108]. Since transcript and protein abundance of CAF1 increase under N depletion and phosphorylation decreases (Figure 4), it appears that phosphorylation inhibits CAF1 recruitment, likely repressing translation of unnecessary mRNA and conserving N during N limitation. BDF1 is also known to play a role controlling transcription through binding to TATA-containing promoter regions [109]. Additionally, BDF1 binds to histones, has a role in histone modification and can affect chromatin structure [110,111]. Phosphorylation of BDF1 has also been found in yeast, where phosphorylated BDF1 was necessary for function and may impact RNA polymerase II transcription [111]. Thus, it is possible that significant dephosphorylation/inhibition of BDF1 under N deplete conditions, plays a role in dampening transcription.

### 3.9. Stress Related Signaling and Kinases Affected by N Status

Phosphorylation of HSP90 (Phatr3_J55230) and manganese superoxide dismutase (MnSOD, Phatr3_J42832) are affected by N. HSP90 is dephosphorylated as cellular N is depleted. In yeast, phosphorylation of HSP90 has been shown to inhibit HSP90 conformational change, affecting DNA repair. Although phosphorylated on both serine and threonine residues, 4 serine residues were identified to be important phosphorylation sites in yeast and dephosphorylation occurred through Ser/Thr phosphatase Ppt1 [112]. However, in *P. tricornutum*, only phosphorylation of threonine was found to be affected by N depletion (Thr-601). Since *P. tricornutum* HSP90 protein abundance increases in response to N depletion, it appears to be dephosphorylated and active during N depletion (Figure 4), similar to yeast HSP90. Mitochondria localized MnSOD detoxifies mitochondrial oxygen free radicals generated during mitochondrial respiration and has been shown to be phosphorylated on Ser106 by Cdk1 in human cells. Phosphorylation of human MnSOD resulted in enhanced enzymatic activity and increased protein stability [113]. Human MnSOD p-site Ser-106 is not conserved in *P. tricornutum*. Since transcript, protein, and phosphopeptide abundance are correlated, increasing during N deplete conditions (Figure 4), it is probable that phosphorylation stabilizes MnSOD and results in increased activity in *P. tricornutum*, promoting ROS scavenging during N depletion.

Phosphorylation and ubiquitination have been shown to overlap, resulting in “crosstalk” in post-translational modification [44]. Phosphopeptides of ubiquitin carboxyl-terminal hydrolase (UCH, Phatr3_J42729) and ubiquitin E3 ligase (UbE3; Phatr3_EG01549) were found in the Nshort-P subset and were correlated to transcript expression (Figure 4). Interestingly, UCH and UbE3 display contrasting patterns. UCH, which deubiquitinates proteins [114,115], was significantly dephosphorylated during N limitation (log2FC = −2.2) while phosphorylation of UbE3, which ubiquitinates proteins, was highest under N limitation (Figure 4). Since studies on phospho-regulation of UCH are sparse and phosphorylation of UbE3 could activate or inhibit [116,117], it is difficult to speculate on the functional significance of activation/deactivation of these two phosphoproteins. However, it is important to note that phosphorylation of UCH and UbE3 likely plays a role in signaling related to managing protein degradation under N limitation.

Kinases were also differentially phosphorylated between N replete and N deplete conditions and may be responsible for phosphorylation of particular phosphoproteins detected in our dataset. These include a calcium dependent protein kinase (CDPK; Phatr3_J25067), a Ca2+/calmodulin-dependent protein kinase (CaMK; Phatr3_EG02294), an inositol hexakisphosphate/ diphosphoinositol-pentakisphosphate kinase (InsP6/PP-IP5K; Phatr3_J46684), and a serine/threonine kinase (MARK3; Phatr3_J8773). Phosphopeptide abundance of CDPK and CaMK was correlated with total protein abundance and they were both part of the N deplete cluster (Figure 4). Since they were upregulated and phosphorylated during N deplete conditions, it would follow that they are necessary and active under this condition. These data suggest that calcium is an important signaling molecule during N depletion. CDPK has also been shown to be involved in plant stress signaling [118]. The kinase InsP6/PP-IP5K is involved in cellular trafficking and can affect vacuole size in plants [119]. In *P. tricornutum*, phosphorylated InsP6/PP-IP5K is anti-correlated to both transcript and total protein abundance (Figure 4), indicating that dephosphorylation/activation of InsP6/PP-IP5K is under N deplete conditions. Similarly, MARK3 also has a contrasting transcript/protein expression pattern. During N replete conditions, transcript and protein abundance of MARK3 are elevated and it is dephosphorylated (Figure 4). Thus, we also hypothesize that MARK3 is dephosphorylated/active during N replete and phosphorylated/inactive during N deplete conditions. N sensitive kinase activity is important as it is believed that phosphorylation triggers the signaling cascade through which the cell senses the presence of nitrogen. Smith et al. (2019) identified a subset of 61 genes that are “highly nitrate responsive” (HNS) which were transcriptionally upregulated in the presence of nitrate. However, we found only one hypothetical protein (Phatr3_J36843) to be common between HNS and Nshort-P subsets. Thus, we propose that kinases identified in the HNS subset (Phatr3_J21961, Phatr3_J16036 and Phatr3_J15229) [18] and kinases identified in the Nshort-P subset (CDPK, CaMK, InsP6/PP-IP5K and MARK3) are the most likely participants in nitrogen sensing.

## 4. Conclusions

Research on diatoms have included the cellular response to nutrient limitation (Si, N, P, Fe) in the context of carbon sequestration, global biogeochemical cycling, and diatom bloom dynamics. From a bioengineering perspective, diatoms have been used to study lipid accumulation for purposes related to fuels, food, and production of high value compounds [120]. We believe that further investigation into post-translational phospho-regulation in response to shifts in nutrient availability will result in increased understanding of key nutrient sensing, signaling, and metabolic mechanisms that control physiological acclimation in diatoms.

Most of the diel light cycling related protein phosphorylation we have identified in this study occur around the transitions between light and dark period. In response to the onset of the light period, under diel light cycling conditions, we observed phosphorylation patterns for key proteins involved in controlling C flux in diatoms. These data suggest an important role of phospho-regulation in shuttling C towards fatty acid biosynthesis. Additional studies that quantitatively track the PEP, pyruvate, and oxaloacetate metabolite pools over the diel cycle in conjunction with the abundance and phosphorylation status of proteins such as Pt_ACC, Pt_PPDK, Pt_MDH, will be helpful. Generation of p-site point mutations for such proteins are an important next step for examining the importance of phospho-regulation for controlling key aspects of C flux within the cell. Moreover, to further grasp the dynamics of global phosphorylation as cells transition between light regimes, a shorter sampling interval would be essential for future studies. Additionally, phosphorylation events related to cell cycle dynamics were not detected, which could be due to minimal growth synchronization of *P. tricornutum* observed in our experiment [18]. Thus, an algal species able to synchronize to light/dark regimes, such as *C. reinhardtii*, could be used for future studies.

Phosphorylation of the key Fe metabolism proteins, flavodoxin and pTF, was shown to differ based on Fe status of the cell. Additional biochemical studies on the role of phosphorylation in regulating these proteins will result in better understanding of the mechanisms by which diatoms sense, scavenge and assimilate Fe in the environment. Additionally, phosphorylation changes between N replete and deplete cellular states discussed in this study provide a new perspective to the mechanisms behind the early diatom response to N depletion. Studies on the transcriptional response of diatoms to the sudden onset NO_3_ availability [18] have shown that a dramatic response requires only a few minutes. According to nitrate transporter studies in plants [93,121], fungi [95,96,122] and bacteria [123], phosphorylation is likely an important trigger for N sensing and kinases, such as MARK3 and HNS kinases, are a likely component of the N sensing. Since the Nshort experiment was only a short time course, phosphorylation comparisons of the early N deplete (18 h) timepoint and a longer N starved sample may have further interesting insights into the role of phosphorylation on stress related mechanisms. Furthermore, a study of how phosphorylation plays a role in the recovery of N deplete cells would also lead to a better understanding of the competitive advantage of diatoms in the environment.

Our results serve to establish new insight into phospho-regulation in the model diatom *P. tricornutum* under low Fe, diel light cycling, and across a transition of N availability. We have shown that C and N flux, stress responses and ubiquitination/deubiquitination are likely under the control of phospho-regulation. Additionally, overall transcriptional output appears to be governed by phosphoproteins that affect binding to TATA regions, histone stability, and chromatin accessibility. Furthermore, our data suggest that phospho-regulation likely plays a role in dictating the diversity and quantity of the cellular protein pool through phosphorylation of translation initiation, translation accuracy and translation elongation proteins. Overall, phosphorylation appears to play a major role in regulating cellular processes and signal cascades. When considered in conjunction with quantitative transcriptomics, metabolomics, and proteomics, additional studies into phosphorylation patterns in response to acute shifts in environmental conditions will likely result in new insights into diatom signaling and metabolic mechanisms that are key for controlling diatom physiological acclimation and growth.

## Supplementary Materials

The following are available online at https://www.mdpi.com/2079-7737/9/7/155/s1. Table S1: Cell concentrations, growth rate and doubling time for Nshort experiments. Table S2: Manually curated phosphoproteome log abundance data. Table S3: Compilation of phosphopeptides detected in *P. tricornutum*, including phosphopeptides also detected in the Chen et al. (2014) study [30]. Table S4: Normalized protein and phosphopeptide abundance data of LowFe-P subset proteins. Table S5: Normalized protein and phosphopeptide abundance data of Diel-P subset proteins. Table S6: Normalized transcript, protein and phosphopeptide abundance data of Nshort-P subset proteins, including log2FC calculations between timepoints. Table S7: Summary of the possible effect of phosphorylation on phosphoproteins. Figure S1: PCA of Nshort transcriptome and proteome samples. Figure S2: Normalized protein and phosphopeptide abundance plots of 7 diel responsive phosphoproteins. Figure S3: Normalized transcript, protein and phosphopeptide plots for flavodoxin (Phatr3_J23658) and ISIP2A (Phatr3_J54465) over the diel cycle at low, medium and high Fe treatments.
