# Supplementary material for: The Importance of Protein Phosphorylation for Signaling and Metabolism in Response to Diel Light Cycling and Nutrient Availability in a Marine Diatom
Source: Biology (Basel). 2020 Jul 6;9(7):155. doi: 10.3390/biology9070155 (PMC7408324; doi:10.3390/biology9070155)
Supplement: Supplementary file 1 [file biology-09-00155-s001.zip › ROUND2_Supp/Table S1 Cell counts.docx]

Table S1: Cell concentrations, growth rate and doubling time for Nshort experiments.

| Sample | 15 min  (cells mL ^-1^) | 45 min  (cells mL ^-1^) | 18 h  (cells mL ^-1^) | μ day ^-1^ | Doubling time  (day) |
| --- | --- | --- | --- | --- | --- |
| N- A | 7.60 x 10^6^ | 7.60 x 10^6^ | 1.12 x 10^7^ | 0.52 | 1.34 |
| NH_4_ B | 5.65 x 10^6^ | 7.95 x 10^6^ | 1.01 x 10^7^ | 0.77 | 0.89 |
| NO_3_ B | 7.48 x 10^6^ | 4.99 x 10^6^ | 1.11 x 10^7^ | 0.53 | 1.32 |
|  |  |  |  |  |  |
| Average | 6.91 x 10^6^ | 6.85 x 10^6^ | 1.08 x 10^7^ | 0.45 | 1.16 |

Note: Data points represent single replicate counts for the pre-treatment, and different N sources (no data for NO2-). Cell counts were done using the disposable hemocytometers, InCyto C-Chip DHC-N01 (Neubauer Improved) glass two-grid slides. A minimum of 200 cells counted, 2X grids, then averaged.
