# Supplementary material for: The Importance of Protein Phosphorylation for Signaling and Metabolism in Response to Diel Light Cycling and Nutrient Availability in a Marine Diatom
Source: Biology (Basel). 2020 Jul 6;9(7):155. doi: 10.3390/biology9070155 (PMC7408324; doi:10.3390/biology9070155)
Supplement: Supplementary file 1 [file biology-09-00155-s001.zip › ROUND2_Supp/Table S7 Phosphorylation_Summary.docx]

| Table S6: Summary of the possible effect of phosphorylation on phosphoproteins. | | | |
| --- | --- | --- | --- |
| **Phosphoprotein** | **Phosphorylation** | **Condition phosphorylated** | **Reference/Evidence** |
| flavodoxin (Phatr3_J23658) | Activates | Low Fe | Transcript upregulated [15] |
| E3 ubiquitin protein ligase (Phatr3_J12887) | Activates | Low Fe | Transcript upregulated [15] |
| ferrocytochrome oxygenase (Phatr3_J12588) | Activates | Low Fe | Transcript upregulated [15] |
| ISIP2A phytotransferrin (Phatr3_J54465) | Activates | - | Transcript upregulated [15] |
| NAD-glutamate dehydrogenase (NAD-GDH; Phatr3_J45239) | Inactivates | Low Fe, N-18h | C and N reallocation [44], decreased activity [98] |
| 2-oxoglutarate dehydrogenase (2-OGDH; Phatr3_J29016), | - | Low Fe | C and N reallocation [44] |
| phosphoglycerate mutase (Phatr3_J45200), | - | Low Fe | C reallocation [44] |
| mannose-6-phosphate isomerase (Phatr3_J10693) | - | Low Fe | C reallocation [44] |
| catalase (Phatr3_J22418) | Activates | Low Fe | C reallocation [44] |
| glycosyl hydrolase (Phatr3_J1372) | Activates | Low Fe | C reallocation [44] |
| FACT complex subunit SSRP1 (Phatr3_EG02355) | Inactivates | Low Fe | Inhibits DNA binding and reduced transcription [45] |
| CCR-associated factor 1 (Phatr3_J9576) | Inactivates | Low Fe, N-15m | Transcript and protein data [18] |
| ribosomal proteins S3 (Phatr3_J17545) | Inactivates | Low Fe | Conformational change [46-50] |
| ribosomal proteins L24 (Phatr3_J19152) | Inactivates | Low Fe | Conformational change [46] |
| elongator complex protein 1 (Phatr3_J43627) | Activates | Low Fe | Wobble uridine tRNA modification [53,54] |
| chloroplast phosphoglycerate kinase (Phatr3_J29157) | Inactivates | Dark-9h | *A. thaliana* p-sites [59] |
| chloroplast glyceraldehyde 3-phosphate dehydrogenase (Phatr3_J22122) | Inactivates | Dark-9h | Lowers Vmax, disrupts binding to 14-3-3 proteins [60] |
| cytoplasmic acetyl-CoA carboxylase (Phatr3_J55209) | Inactivates | Dark-9h | Mouse p-sites [61,62] |
| chloroplast pyruvate orthophosphate dikinase (Phatr3_J21988) | Inactivates | Dark-9h,  Light-1h | *Z. mays* p-sites [65,66] |
| mitochondrial NAD-dependent malate dehydrogenase (Phatr3_J42398) | Inactivates | Dark-9h | *S. cerevisiae* p-sites, shows degradation [67] |
| plasma membrane localized urea symporter (Phatr3_J20424) | Inactivates | Light, N-15m | Low urea cycle activity [15,69] |
| cytoplasmic glutamine-dependent carbamoyl phosphate synthetase (Phatr3_EG01947) | Inactivates | Transition,  N-15m | Transcript upregulated [15], protein accumulates in the dark [69], human p-sites [99,100] |
| vacuolar sorting-associated protein (Phatr3_EG02146) | - | Light-1h | - |
| small ribosomal subunit 40 S9 (Phatr3_J6847) | - | Dark-9h | *A. thaliana* [72,73], human p-sites [74] |
| 60S acidic ribosomal protein P0 (Phatr3_J30660) | - | Light-9h | Ribosome activity and regulation [76] |
| elongation factor 3A (Phatr3_EG02323) | - | Dark-9h | Translation elongation and translational accuracy [77,78] |
| eukaryotic elongation factor 2 kinase (Phatr3_EG02345) | - | Light-1h | Translation elongation and translational accuracy [79-81] |
| eukaryotic translation initiation factor eIF4F (Phatr3_J44383) | - | Light-9h | TOR signaling [82,83] |
| eukaryotic translation initiation factor eIF4G3 (Phatr3_EG02122) | - | Transition | TOR signaling [82,83] |
| oxygen-evolving enhancer protein psbO (Phatr3_J20331) | - | N-15m | - |
| phosphofructokinase (Phatr3_EG02209) | Inactivates | N-15m | Transcript and protein data [18] |
| nitrate/nitrite transporter (Phatr3_J2171) | Inactivates | N-15m | Increased scavenging under N deplete conditions [94,95] |
| ubiquitin carboxyl-terminal hydrolase (Phatr3_J42729) | - | N-15m | Manages protein degradation [42] |
| ubiquitin E3 ligase (Phatr3_EG01549) | - | N-18h | Manages protein degradation [42] |
| Tyr-tRNA ligase (Phatr3_EG02310) | - | N-15m, N-18h | - |
| heat shock protein 90 (Phatr3_J55230) | Inactivates | N-15m | Inhibits DNA repair [110] |
| Ca2+/calmodulin-dependent protein kinase (Phatr3_EG02294) | Activates | N-15m, N-18h | Transcript and protein data [18] |
| calcium dependent protein kinase (Phatr3_J25067) | Activates | N-18h | Transcript and protein data [18] |
| serine/threonine kinase (Phatr3_J8773) | Inactivates | N-18h | Transcript and protein data [18] |
| inositol hexakisphosphate/ diphosphoinositol-pentakisphosphate kinase (Phatr3_J46684) | Inactivates | N-15m | Transcript and protein data [18] |
| 40S small ribosomal subunit 20 (Phatr3_J51291) | - | N-15m | Regulatory feedback loop controlled by phosphorylation [101] |
| translation initiation factor eIF2 gamma (Phatr3_J42307) | Inactivates | N-18h | Research on homolog eIF2a [102-104] |
| gamma-aminobutyric acid type B receptor (Phatr3_Jdraft1756) | Activates | N-18h | Protein upregulated [18] |
| phosphoenolpyruvate carboxykinase (Phatr3_EG02232) | Inactivates | N-18h | Decreases substrate affinity [96,97] |
| manganese superoxide dismutase (Phatr3_J42832) | Activates | N-18h | Increased activity and protein stability [111] |
| cytochrome C oxidase subunit 6b (Phatr3_J11016) | - | N-15m, N-18h | - |
| ammonium-dependent carbamoyl-phosphate synthetase (Phatr3_J24195) | Inactivates | N-18h | Transcript and protein data [18] |
| transcription initiation factor TFIID subunit BDF1 (Phatr3_J44399) | Activates | N-15m | Impacts RNA polymerase II transcription [109] |
| histone deacetylase (Phatr3_J13057) | - | N-18h | - |
| centromere (Phatr3_J43019) | - | N-18h | - |
| sister chromatid cohesion protein (Phatr3_Jdraft1590) | Activates | N-18h | Protein upregulated [18] |
| ammonium transporter AMT1 (Phatr3_J27877) | Activates | N-18h | Transcript and protein data [18] |
