# Supplementary figures and images for: The Importance of Protein Phosphorylation for Signaling and Metabolism in Response to Diel Light Cycling and Nutrient Availability in a Marine Diatom
Source: Biology (Basel). 2020 Jul 6;9(7):155. doi: 10.3390/biology9070155 (PMC7408324; doi:10.3390/biology9070155)

# Nshort Transcriptome

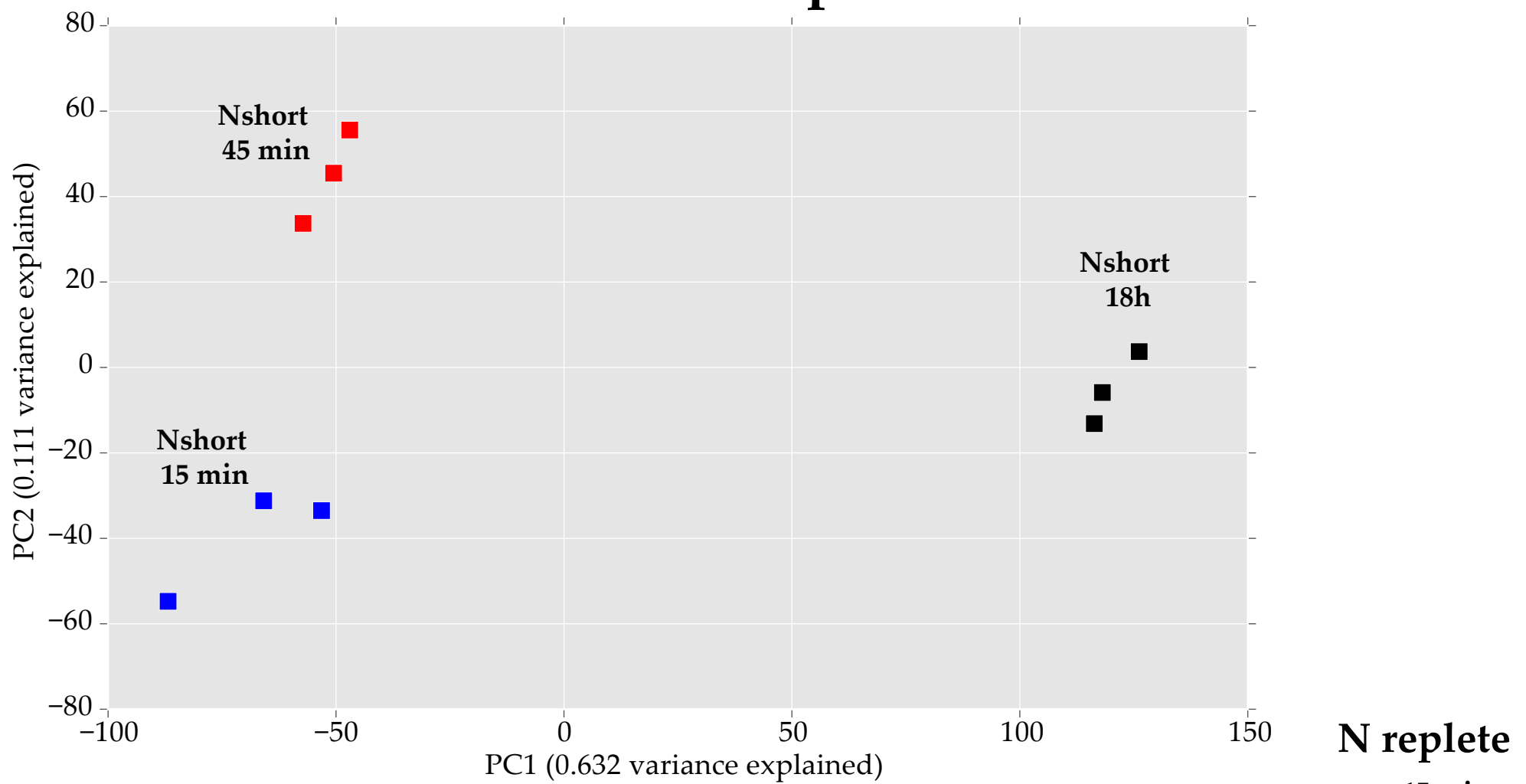

# Nshort Proteome

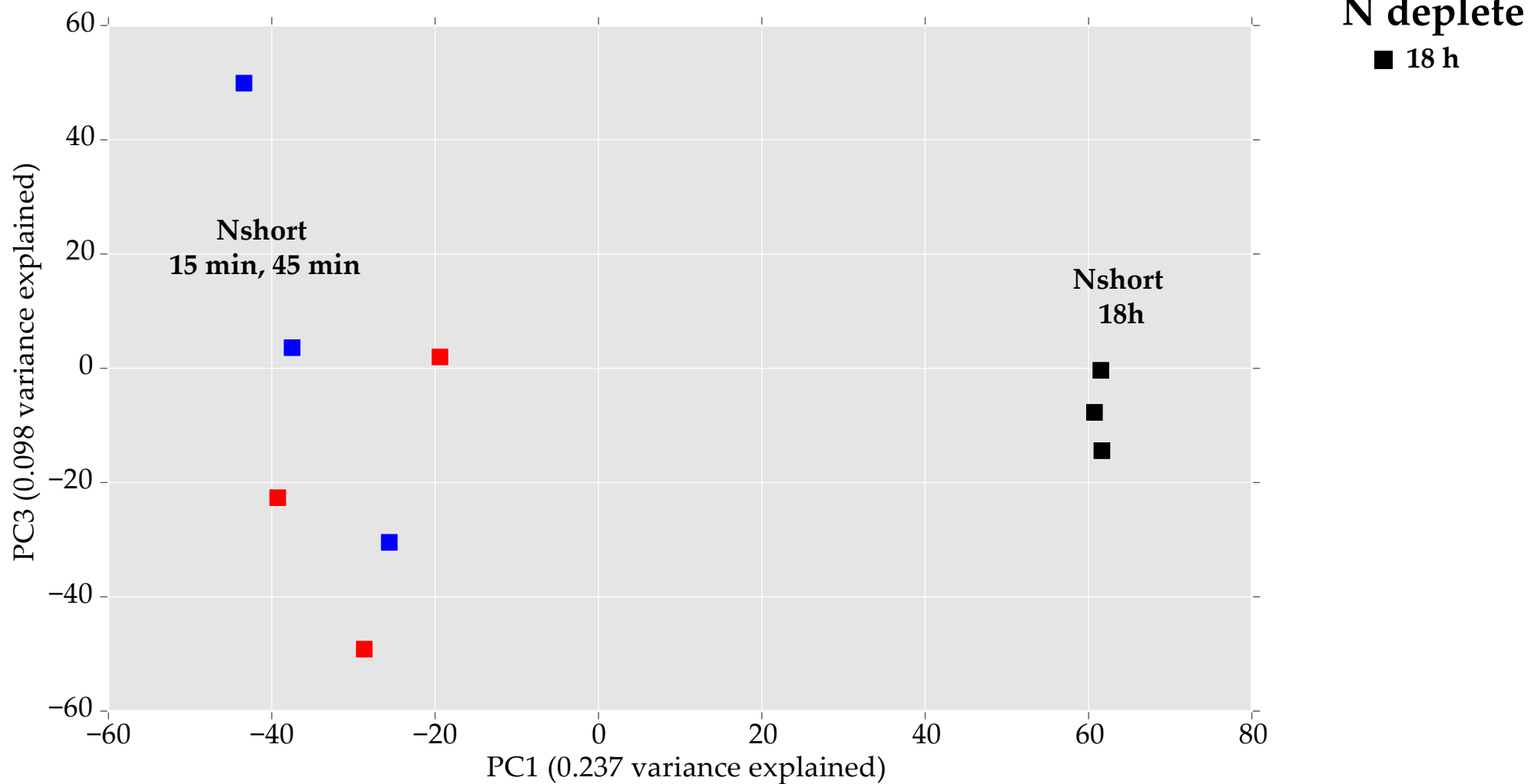

Supplement: Supplementary file 1 [file biology-09-00155-s001.zip › ROUND2_Supp/Figure S1.pdf]

**DUR3**

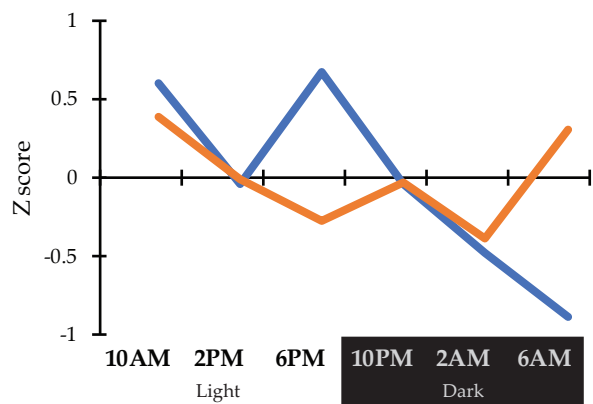

**P0**

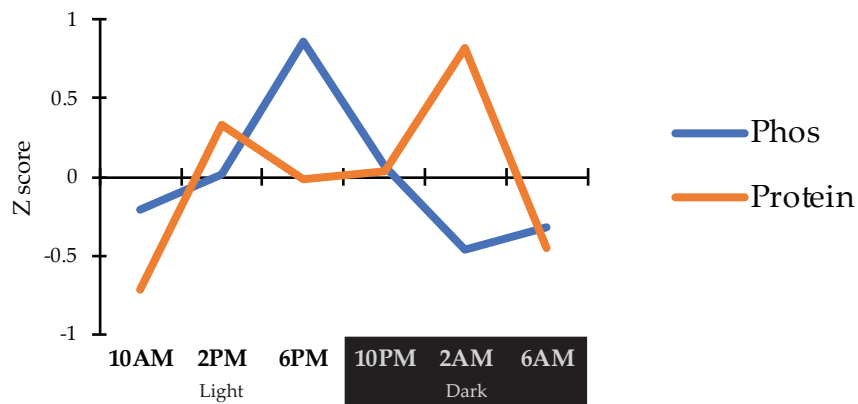

**pgCPS**

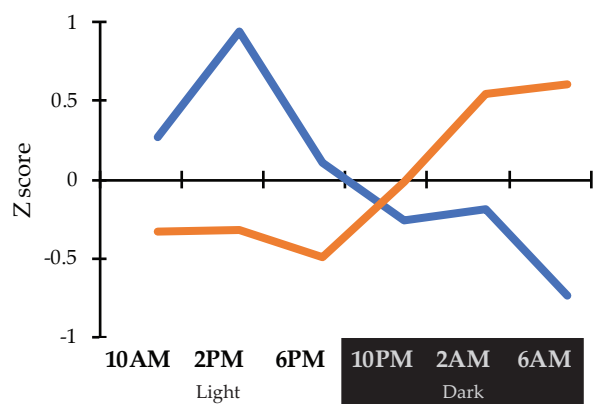

**EF3A**

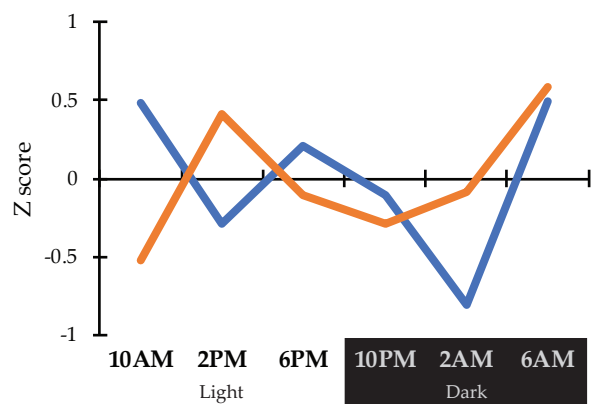

**VSP**

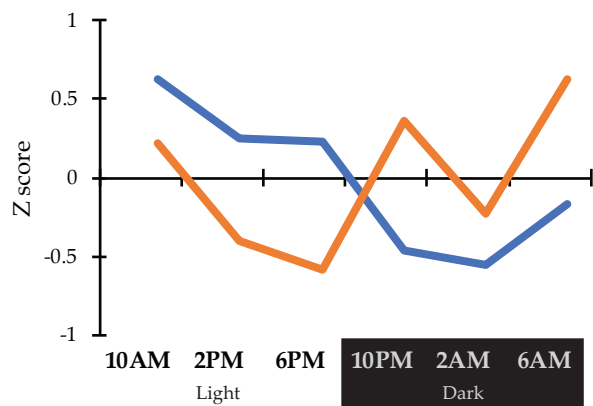

**eEF2K**

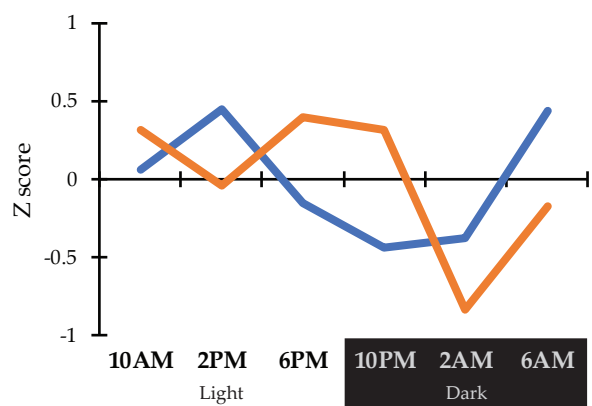

**S9**

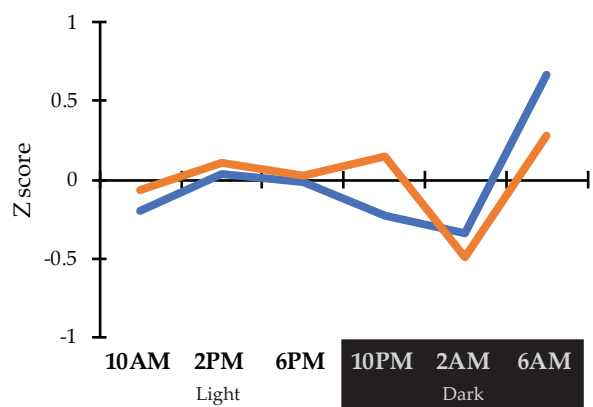

Supplement: Supplementary file 1 [file biology-09-00155-s001.zip › ROUND2_Supp/Figure S2.pdf]

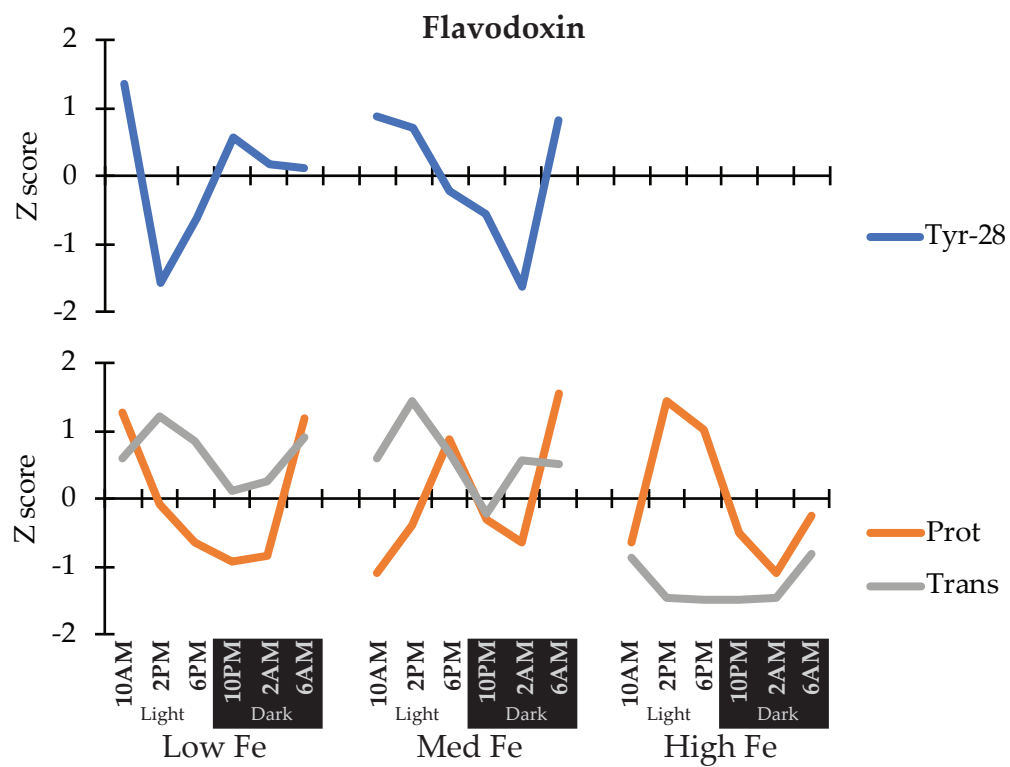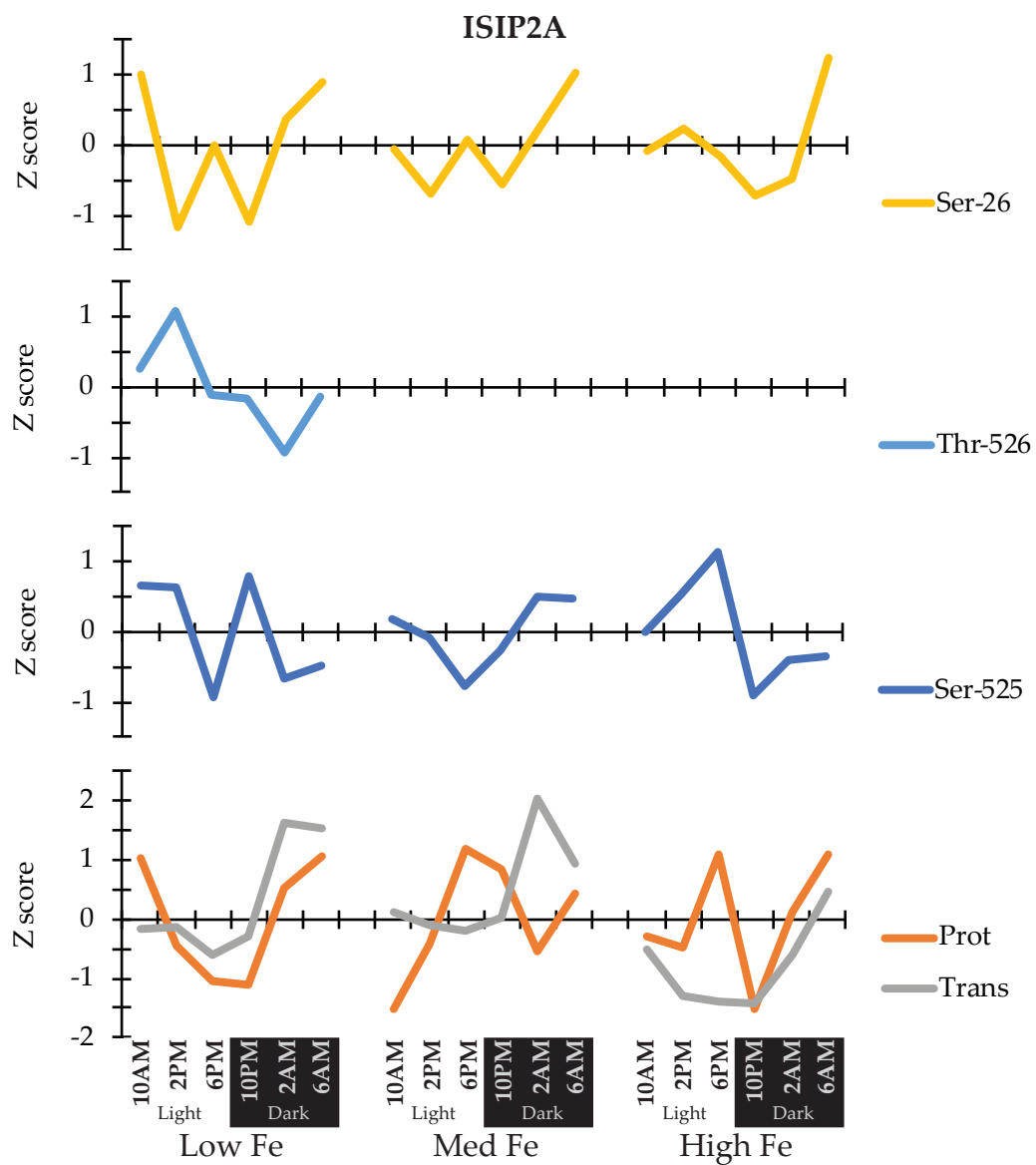

Supplement: Supplementary file 1 [file biology-09-00155-s001.zip › ROUND2_Supp/Figure S3.pdf]
